# Three‐dimensional reconstruction of soybean nodules provides an update on vascular structure

**DOI:** 10.1002/ajb2.1249

**Published:** 2019-03-12

**Authors:** David Livingston, Tan Tuong, Marco Nogueira, Thomas Sinclair

**Affiliations:** ^1^ U. S. Department of Agriculture Raleigh NC USA; ^2^ Brazillian Agricultural Research Corporation Brasilia DF Brazil; ^3^ Department of Crop Science North Carolina State University Raleigh NC USA

**Keywords:** *Glycine max*, roots, infected zone, sclereid layer, soybean, 3D reconstruction, vascular bundles, xylem

## Abstract

**Premise of the Study:**

In many cases, the functioning of a biological system cannot be correctly understood if its physical anatomy is incorrectly described. Accurate knowledge of the anatomy of soybean [*Glycine max* (L.) Merril] nodules and its connection with the root vasculature is important for understanding its function in supplying the plant with nitrogenous compounds. Previous two‐dimensional anatomical observations of soybean nodules led to the assumption that vascular bundles terminate within the cortex of the nodule and that a single vascular bundle connects the nodule to the root. We wanted to see whether these anatomical assumptions would be verified by digitally reconstructing soybean nodules in three dimensions.

**Methods:**

Nodules were dehydrated, embedded in paraffin, and cut into 15 μm thick sections. Over 200 serial sections were stained with safranin and fast green, and then photographed using light microscopy. Images were digitally cleared, aligned, and assembled into a three‐dimensional (3D) volume using the Adobe program After Effects.

**Key Results:**

In many cases, vascular bundles had a continuous connection around the nodules. The 3D reconstruction also revealed a dual vascular connection originating in the nodule and leading to the root in 22 of the 24 nodules. Of the 22 dual connections, 11 maintained two separate vascular bundles into the root with independent connections to the root vasculature.

**Conclusions:**

A more robust and complex anatomical pathway for vascular transport between nodules and root xylem in soybean plants is indicated by these observations and will contribute to a better understanding of the symbiotic relationship between soybean plants and nitrogen‐fixing bacteria within the nodules.

Nodules that support symbiotic nitrogen fixation are sophisticated structures with fixation taking place in the interior of the nodule that is surrounded by an inner cortex. The inner cortex consists of a continuous layer of closely spaced cells and regulates oxygen flux into the nodule interior. This regulation of oxygen is important because nitrogenase in nitrogen‐fixing cells is labile in the presence of even small concentrations of oxygen (Minchin, 1997). The permeability of the inner cortex to oxygen appears to be regulated by air spaces so that the oxygen requirements of the nodule interior are met, but reactive oxygen concentrations in the nodule interior are avoided. An important feature of the nodule interior is the presence in the bacteroid‐infected cells of leghemoglobin that attaches to oxygen at the cell wall of the nitrogen‐fixing cells. This attachment of infected cells to oxygen establishes a diffusion gradient for oxygenated leghemoglobin to the cell interior (Appleby, 1984).

Outside the inner cortex is the outer cortex. Within the loosely packed cells of the outer cortex are the vascular bundles that connect the nodule with the root and the rest of the plant. Materials important for metabolism of the nodule flow to the nodule in the phloem, which is also the main conduit for the supply of water to nodules (Sinclair and Nogueira, 2018). The products of nitrogen fixation are loaded into the xylem and transported to the plant. These nitrogen products must diffuse from the site of fixation in the nodule interior to the vascular bundles and there be actively loaded into the xylem vessels. Failure to readily remove nitrogen products from the nodule can result in nitrogen‐feedback inhibition of nitrogen fixation (Vadez et al., 2000; King and Purcell, 2005; Sulleman et al., 2010).

The structure of the soybean [*Glycine max* (L.) Merril; Fabaceae] nodule vascular system that accounts for flow of water and organic material into and out of the nodule was described in detail by Walsh et al. (1989). They concluded that there was no continuity in the vascular system completely around the nodule but that each bundle terminated in the cortex independent of other bundles. However, this view was subsequently altered by Walsh et al. (1992) in observations that indicated there was vascular continuity in the vascular bundles around the soybean nodule. Walsh et al. (1989, p. 397) further concluded that the vascular bundles coalesced in the nodule near the point of the connection to the root to form “a single vascular strand which had connected the root stele to the nodule”. However, the techniques used by Walsh et al. (1989) to visualize the nodule vascular structure involved chemical digestion and dissection of the nodule to isolate the vascular bundles. They admitted that their procedure “did not allow the pattern of vascularization of a whole nodule to be visualized” (p. 396) and suggested that a three‐dimensional (3D) reconstruction of the nodule vasculature based on serial cross sections would be useful to fully document the nature of the nodule vascular system. The objective of the present study was to develop 3D constructs of the soybean nodule to better understand anatomical features of this important tissue.

## MATERIALS AND METHODS

Soybean cultivar Hutcheson and genotype PI 471938 were grown in a greenhouse (Phytotron, North Carolina State University, Raleigh, NC, USA) with air temperature adjusted to 31°C day/26°C night. Polyethylene pots with 2 L volume were filled with a mixture of steam‐sterilized substrate composed of 50% peat‐moss growing mix (Redi Earth, W.R. Grace Co., Columbia, MD, USA) and 50% cement sand. Five seeds of each genotype, previously inoculated with *Bradyrhizobium* (Nitragin, Brookfield, WI, USA) to promote nodulation, were sown per pot. Plants were irrigated every day with distilled water and once a week with a complete nutrient solution. Five days after shoots emerged from the growing medium, plants were thinned to one per pot and grown to approximately the V5 stage (Fehr and Caviness, 1977). Twenty‐five days after seedlings emerged from the growing medium, nodules were harvested and prepared for microscopic sectioning.

**Figure 1:**
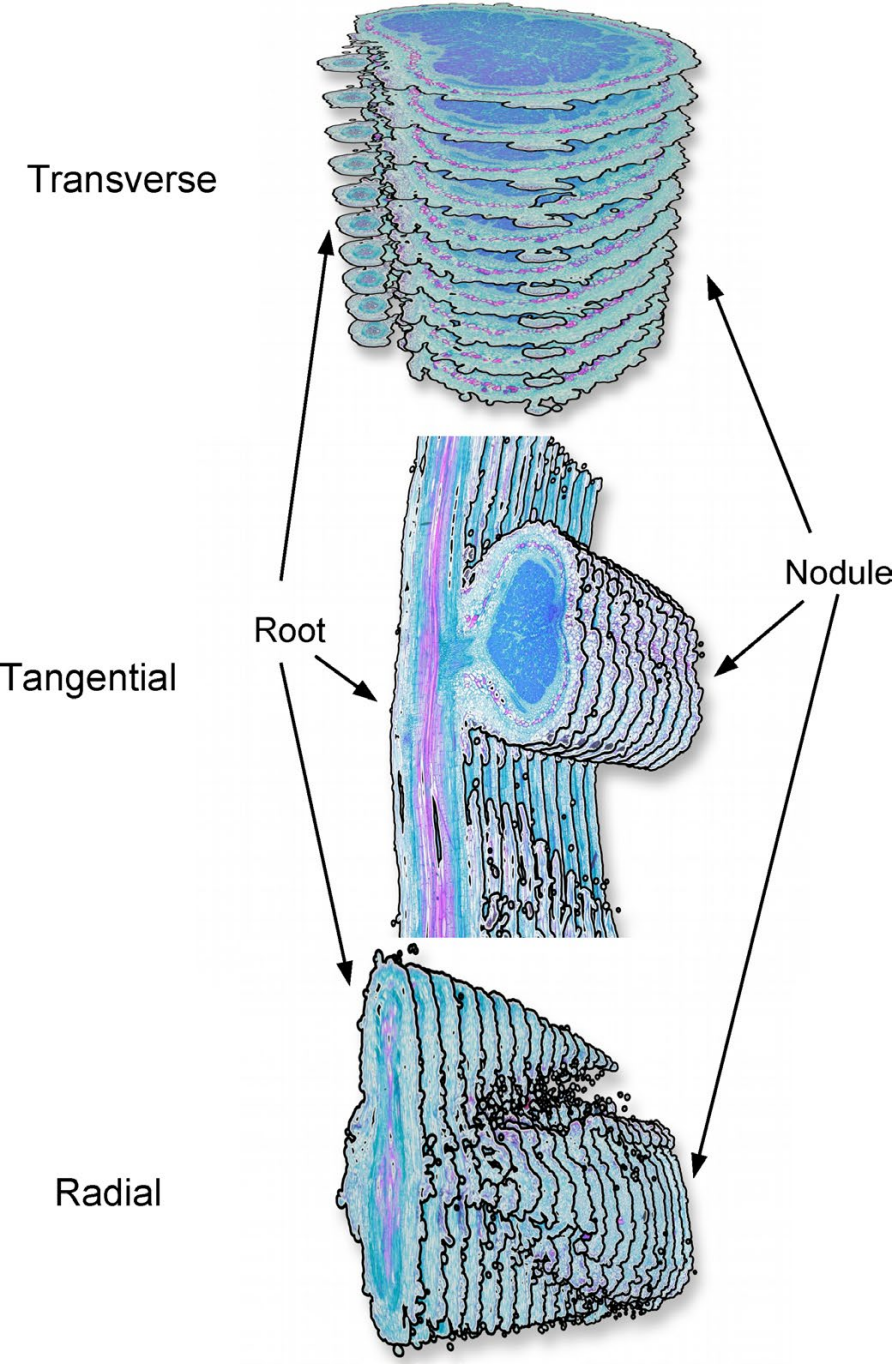
Representative sections showing the three planes in which soybean nodules were sectioned. These are 10 to 20 sequential, 15‐μm‐thick sections taken midway within a series of 200–250 sections. The overall shape of roots and nodules are somewhat distorted owing to the limited number of sections and exaggerated separation between them. The transverse orientation was used in the 3D reconstruction shown in Fig. 2 and Appendix S1. The tangential view was used in Fig. 3 and the radial in Fig. 4. Sections in the tangential and radial views were cut longitudinally with respect to the root, but the radial was rotated 90° (from the tangential) along the root axis.

**Figure 2:**
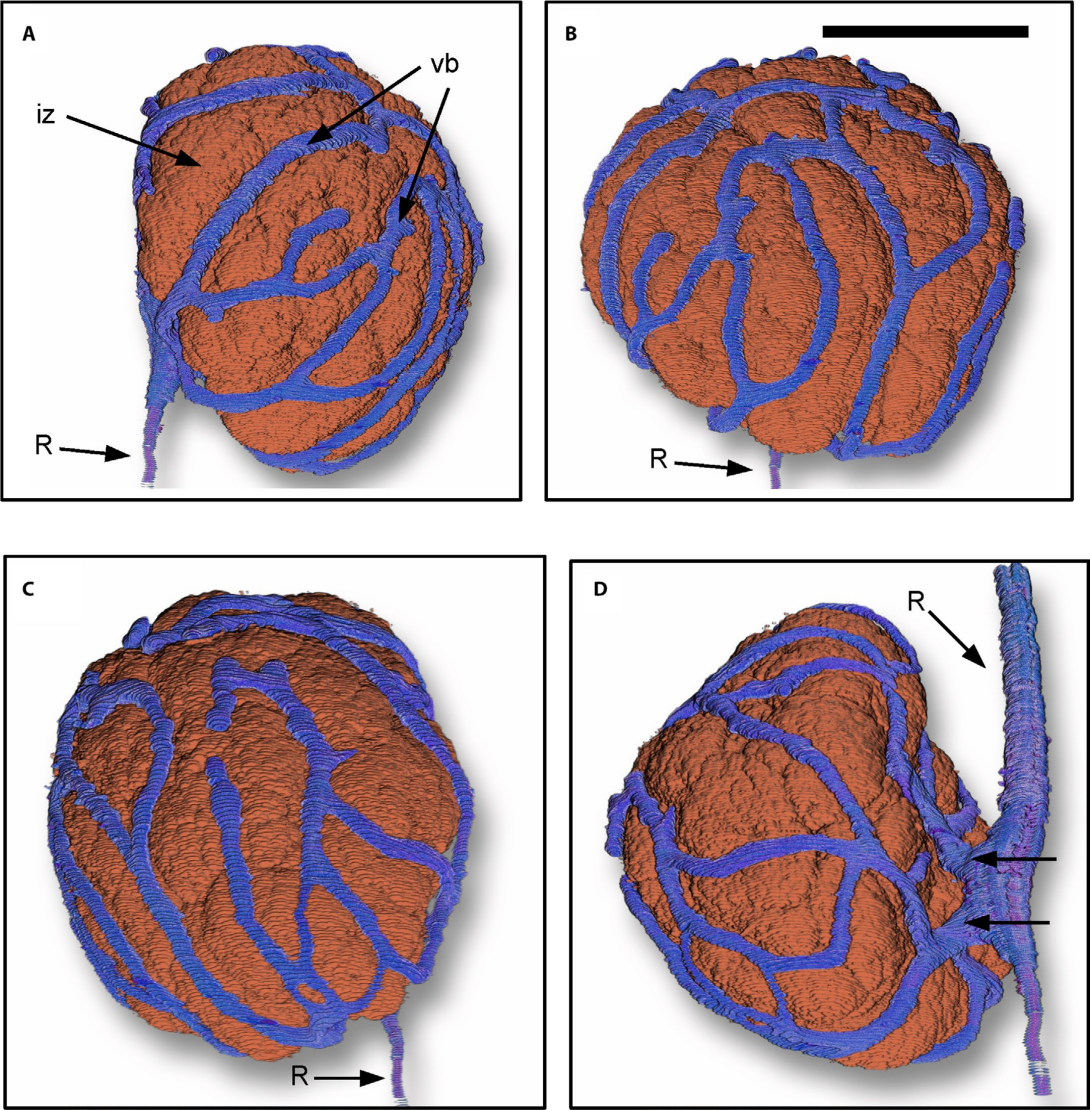
Four views of a 3D reconstruction of infected zone (iz) of a soybean nodule and the vascular bundle (vb). “R” signifies the vasculature of the root. Note the continuity of the vascular system surrounding the infected zone; this continuity is much easier to see in the video in Appendix S1. Also note the dual vascular connection to the root in panel D (arrows). See Appendix S1 for a 3D reconstruction of the complete nodule. The 253 sequential sections used to reconstruct the nodule were cut transversely (Fig. 1). Scale bar: B, 1 mm.

Twelve nodules of each genotype for a total of 24 nodules were processed for sectioning. Nodules and attached roots were removed and placed in formalin—acetic acid–methanol (FAA) fixative (Livingston et al., 2009) at room temperature. The samples were then subjected to a microwave processing technique that involved stepwise dehydration using ethanol (Livingston et al., 2014). Dehydrated nodules and roots were then embedded in Paraplast Plus using a microwave with concomitant vacuum. The embedded tissues were sectioned (15 μm thick) longitudinally with respect to the root to reveal a tangential (4 nodules) and radial surface (15 nodules) showing the root–nodule interface (Fig. 1). In addition, images from transversely sectioned nodules (5 nodules) were used in 3D reconstructions (Fig. 2; Appendix S1). Sections were stained with safranin O and fast green FCF and coverslips affixed with Permount.

Serial sections of stained nodules were used to reconstruct a 3D volume of the nodule (Fig. 2; Appendix S1). Images of sections were captured with a Canon Rebel T5i camera (Canon, Tokyo, Japan) attached to a Nikon Elipse 50i microscope (Nikon, Tokyo, Japan). The images were imported into After Effects (Adobe Systems, San Jose, CA, USA) and digitally cleared to reveal internal structures within the nodule including the vascular system using a technique previously described (Livingston et al., 2014).

## RESULTS

No difference in nodule anatomy was observed between the two genotypes. The 2D sections supported the general observations of Walsh et al. (1989) to some extent. However, the 3D constructs revealed some important differences in nodule vascular structure than was suggested by Walsh et al. (1989). As discussed below, many of the vascular bundles did not terminate in the cortex but formed a continuum of vascular connection completely around the nodule (Fig. 2A–C; Appendix S1). In addition, in about half of the nodules, the nodule was connected to the vascular tissue of the root by two distinct vascular strands (Fig. 2D).

### Vascular continuity

The continuity or termination of the vascular bundles was difficult to visualize in 2D images. From reconstructing the nodule in 3D, it was clear that while some vascular strands did terminate in various regions of the cortex, many of the vascular strands encircled the entire nodule as seen from the different perspectives obtained from the 3D results shown in Fig. 2 and in Appendix S1. The continuity of the vascular strands is unmistakable in the Appendix S1 video of a nodule from Hutcheson in which the nodule is rotated so that the individual vascular bundles can be readily tracked. A similar pattern of vascular continuity around the nodule was seen in a nodule from soybean cultivar PI 471938 (not shown).

### Dual nodule–root connection

**Figure 3:**
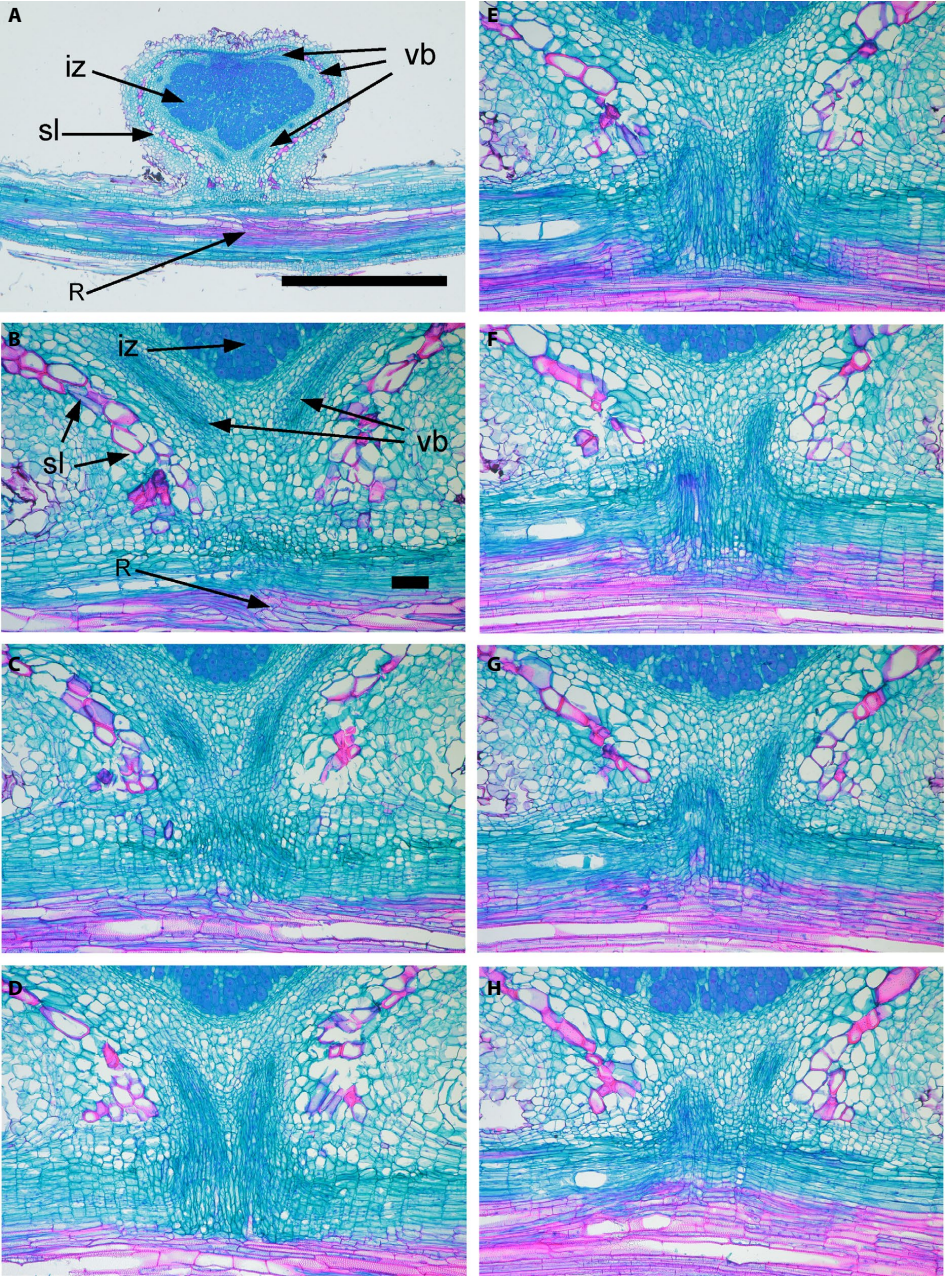
Tangential view (Fig. 1) of the vascular connection between a soybean root and nodule. (A) Low magnification, near the median section of the nodule and root. iz, infected zone; vb, vascular bundles within the cortex and surrounding the infected zone; sl, sclereid layer; R, vasculature within the root. (B–H) Near‐sequential sections (every third section) showing the point of attachment; labels in B also apply to C–H. Note the dual connection all the way to the root. Scale bars: A, 1 mm; B, 100 μm (for C–H also). Bright‐field optics, safranin, fast green staining.

**Figure 4:**
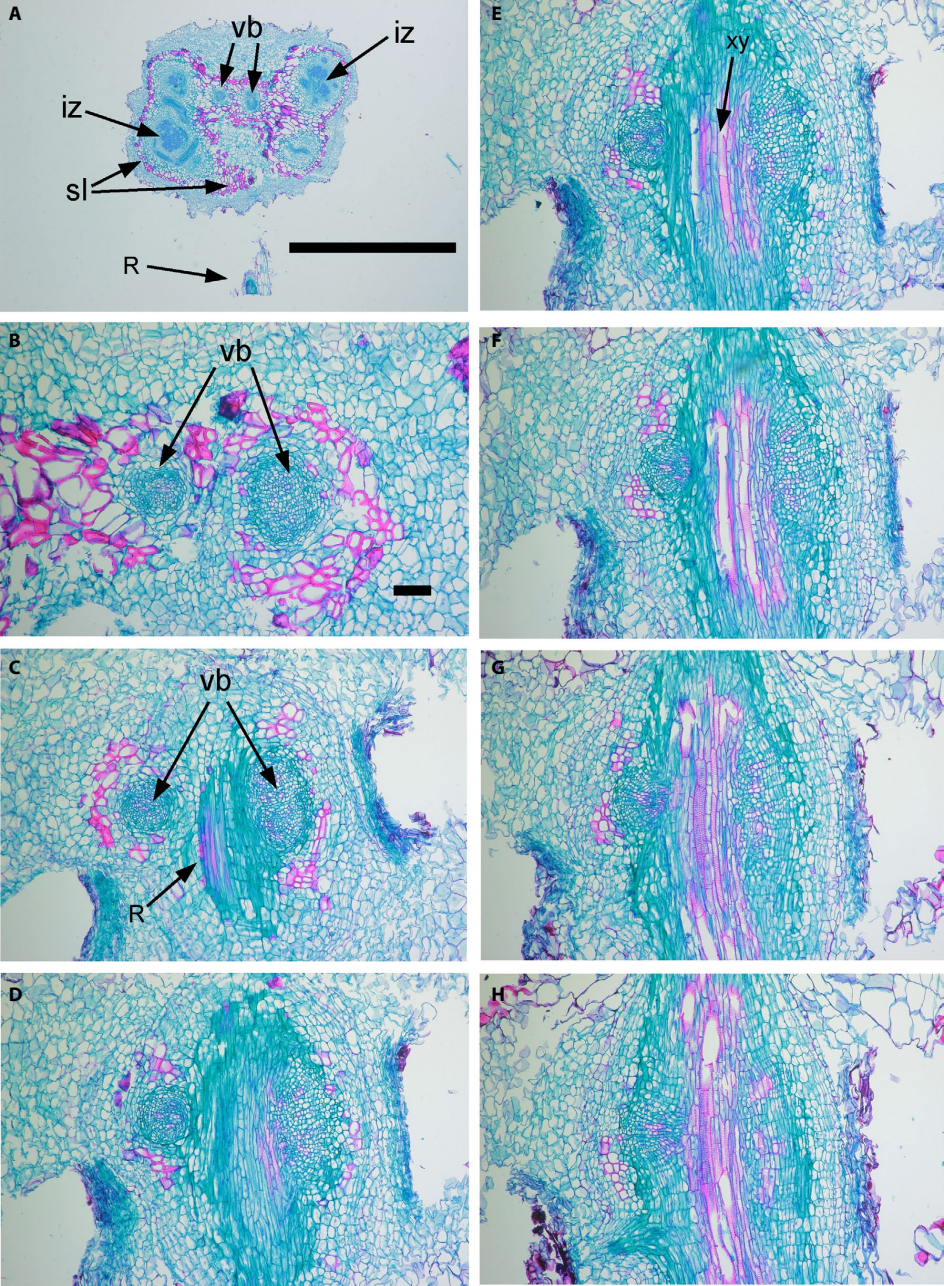
Radial view (Fig. 1) of the vascular connection between a soybean root and nodule. Note the dual connection within the nodule that is continuous all the way to the root. In this instance, the point of attachment of the vascular bundle from the nodule was on either side of the root rather than along the axis of the root as in Fig. 2 and in Appendix S1 and Fig. 3. (A) Low magnification of nodule with the root (R) just coming into view. iz, infected zone; vb, the two vascular bundles that connect to the root. (B) Higher magnification of the two bundles shown in A. Note the gradual disappearance of the roughly circular vascular bundles of the nodule as they merge with the vasculature of the root in B to H. The “R” in panel C shows the first contact with the root made by the vascular bundle on the right side; “xy” in E shows xylem vessels in the root. Labeling in B, C, and E also applies to D, F–H. Scale bar: B, 100 μm (for C–H also). Bright‐field optics, safranin, fast green staining.

Initial observation of 2D images of the connection to the root appeared to indicate a single vascular connection between the nodule and root as reported by Walsh et al. (1989). It was not until transversely sectioned nodules (Fig. 1) were reconstructed in three dimensions that a dual nodule–root connection was discovered (Fig. 2D; Appendix S1). In fact, a continuous single connection between the nodule and root was observed in only two of the 24 nodules. The existence of the dual vascular bundles leading from the nodule was clearly visible in both tangential (Figs. 1, 3) and radial sections (Figs. 1, 4). In half of the 22 nodules with two vascular bundles exiting the nodule cortex (Fig. 3A, B), the two bundles remained separate throughout the nodule–root connection and converged with the root xylem at two separate sites (Fig. 3H). In the remaining 11 nodules with dual vascular bundles exiting the nodule cortex, the two bundles merged with each other at different locations in the nodule–root connection. The point where the two vascular bundles merged occurred at various positions; some merged at the very edge of the nodule, some midway, and some just before connecting to the root (not shown).

## DISCUSSION

Walsh et al. (1989) offered a description of nodule vascular structure that has been the key reference even though they suggested that a 3D construction would be a “useful” addition. In fact, the 3D reconstruction presented in this paper (Appendix S1) showed that the vascular structure is more complex than suggested by Walsh et al. (1989). The key points of difference with Walsh et al. (1989) are (1) that there is a continuity of many vascular strands in the cortex around the entire nodule, and (2) in the vast majority of cases, two vascular strands were found in the connection between the nodule and root.

The continuity of vascular strands around the nodule and the dual connection between the nodule and root suggests the presence of a more robust means of transporting water and organic materials to and from the nodule. Instead of “dead‐end” vascular bundles existing in the nodule cortex, much of the nodule is serviced by the phloem and xylem that has the option for two directions of flow. Hence, a restricted flow in the vascular bundle in one direction may be overcome by flow in the opposite direction. This dual possibility for vascular flow may help to avoid isolation of specific segments of the nodule and allow more uniform nitrogen fixation throughout the nodule.

A dual vascular connection to the root is not unlike what was reported for pea (*Pisum sativum* L.) (Pepper et al., 2007). Our observation that two vascular strands enter the nodule–root connection from the nodule, as in pea, offers a redundancy in the critical flow point in and out of the nodule. In nodules that had two sites of connection with the vascular bundles of the roots, the possibility of flow into and out of the nodule is greater than seems likely with a single vascular strand between the nodule and root. Similar to Walsh et al. (1989), we observed only 8 to 16 tracheary elements of about 8 μm diameter in the vascular strand, but the existence of two vascular strands between the nodule and root indicates a much less constrained flow than anticipated by the original view of a single strand connection. Hence, the capacity for flow between the nodule and root in many cases appears to be about double what could be expected with the original model of Walsh et al. (1989).

These new observations of vascular structure are not, however, inconsistent with the functioning of the vascular flow in support of nodule activity suggested by Walsh et al. (1989). That is, the new view of the vascular structure allows for flow of water and organic materials through the phloem into and around the nodule and for the flow of water and nitrogen products of nitrogen fixation from the nodule through the xylem. These flows are established by hydrostatic gradients that would be fully operational even though there is a continuity of the vascular bundles around the nodule. The presence of dual vascular bundles from the nodule into the connection between the nodule and root offers a redundant and possibly expedited flow into and out of the nodule. These new observations indicate a more robust vascular structure to support nitrogen fixation in nodules.

## Supporting information


**APPENDIX S1 (VIDEO).** Three dimensional reconstruction of a nodule from Hutcheson soybean. The transparency of the outer layers of the nodule was increased gradually in the video to allow visualization of internal structures. Note the vascular system surrounding the infected zone toward the end of the video that shows continuity of most of the vascular bundles. Also note the dual connection of the vascular system as it merges with the vasculature of the root. The 253 15‐μm‐thick sections were digitally cleared and aligned and the 3D volume animated within After Effects.
